# Association of perioperative blood pressure with long-term survival in rectal cancer patients

**DOI:** 10.1186/s40880-016-0100-8

**Published:** 2016-04-11

**Authors:** Hui-Chuan Yu, Yan-Xin Luo, Hui Peng, Xiao-Lin Wang, Zi-Huan Yang, Mei-Jin Huang, Liang Kang, Lei Wang, Jian-Ping Wang

**Affiliations:** Department of Colon and Rectum Surgery, The Sixth Affiliated Hospital (Guangdong Gastrointestinal and Anal Hospital), Sun Yat-sen University, Guangzhou, Guangdong 510655 P. R. China; Guangdong Provincial Key Laboratory of Colorectal and Pelvic Floor Disease, The Sixth Affiliated Hospital (Guangdong Gastrointestinal and Anal Hospital), Sun Yat-sen University, Guangzhou, Guangdong 510655 P. R. China

**Keywords:** Blood pressure, Radical surgery, Rectal cancer, Cancer-specific survival

## Abstract

**Background:**

Several studies suggested that hypertension is positively related to cancer incidence and mortality. In this study, we investigated the association between perioperative blood pressure (BP) and long-term survival outcomes in patients with rectal cancer.

**Methods:**

This study included a cohort of 358 patients with stages I–III rectal cancer who underwent a curative resection between June 2007 and June 2011. Both pre- and postoperative BPs were measured, by which patients were grouped (low BP: <120/80 mmHg; high BP: ≥120/80 mmHg). The survival outcomes were compared between these two groups. The primary endpoints were disease-free survival (DFS) and cancer-specific survival (CSS).

**Results:**

Univariate analysis showed that patients with high preoperative systolic BP had lower 3-year DFS (67.2% vs. 82.1%, *P* = 0.041) and CSS rates (81.9% vs. 94.8%, *P* = 0.003) than patients with low preoperative systolic BP, and the associations remained significant in the Cox multivariate analysis, with the adjusted hazard ratios equal to 1.97 [95% confidence interval (CI) = 1.08–3.60, *P* = 0.028] and 2.85 (95% CI = 1.00–8.25, *P* = 0.050), respectively. Similarly, in postoperative evaluation, patients with high systolic BP had significantly lower 3-year CSS rates than those with low systolic BP (78.3% vs. 88.9%, *P* = 0.032) in univariate analysis. Moreover, high pre- and/or postoperative systolic BP presented as risk factors for CSS in the subgroups of patients who did not have a history of hypertension, with and/or without perioperative administration of antihypertensive drugs.

**Conclusions:**

High preoperative systolic BP was an independent risk factor for both CSS and DFS rates, and high postoperative systolic BP was significantly associated with a low CSS rate in rectal cancer patients. Additionally, our results suggest that rectal cancer patients may get survival benefit from BP control in perioperative care. However, further studies should be conducted to determine the association between BP and CSS and targets of BP control.

## Background

Colorectal cancer is the third leading cause of cancer-related death in the United States [1] and the fifth leading cause of cancer-related death in China, and its annual incidence is increasing [2, 3]. Therefore, many clinical and basic research studies have focused on the development of novel biomarkers to predict treatment outcomes and ensure optimal treatment allocation for patients with colorectal cancer [4]. Because of the increasing longevity rates and the increasing prevalence of obesity, hypertension has become a leading global disease [5]. A meta-analysis demonstrated a positive association between hypertension and all-site cancer incidence and mortality [6]. Moreover, many studies have reported this association for specific cancers, including renal carcinoma, which is the most common site-specific cancer [7]. Additional studies have shown that colorectal cancer risk is positively related to elevated blood pressure (BP) [8–12]. However, these findings were inconsistent, and in previous studies patients’ BP was measured only at the time of baseline health examination. Furthermore, existing data are hampered by lack of adjustment for some potential confounding factors, such as tumor stage, tumor location, and the tumor’s histologic features.

Against this background of inconsistent results and study limitations, we extended these findings by examining the association of perioperative BP with long-term survival outcomes in rectal cancer patients treated with radical-intent surgery. If a positive association does exist, efforts to control BP in perioperative care may lead to extended survival for these patients, which may in turn reveal new therapeutic targets and prognostic biomarkers.

## Patients and methods

### Patients

**Fig. 1:**
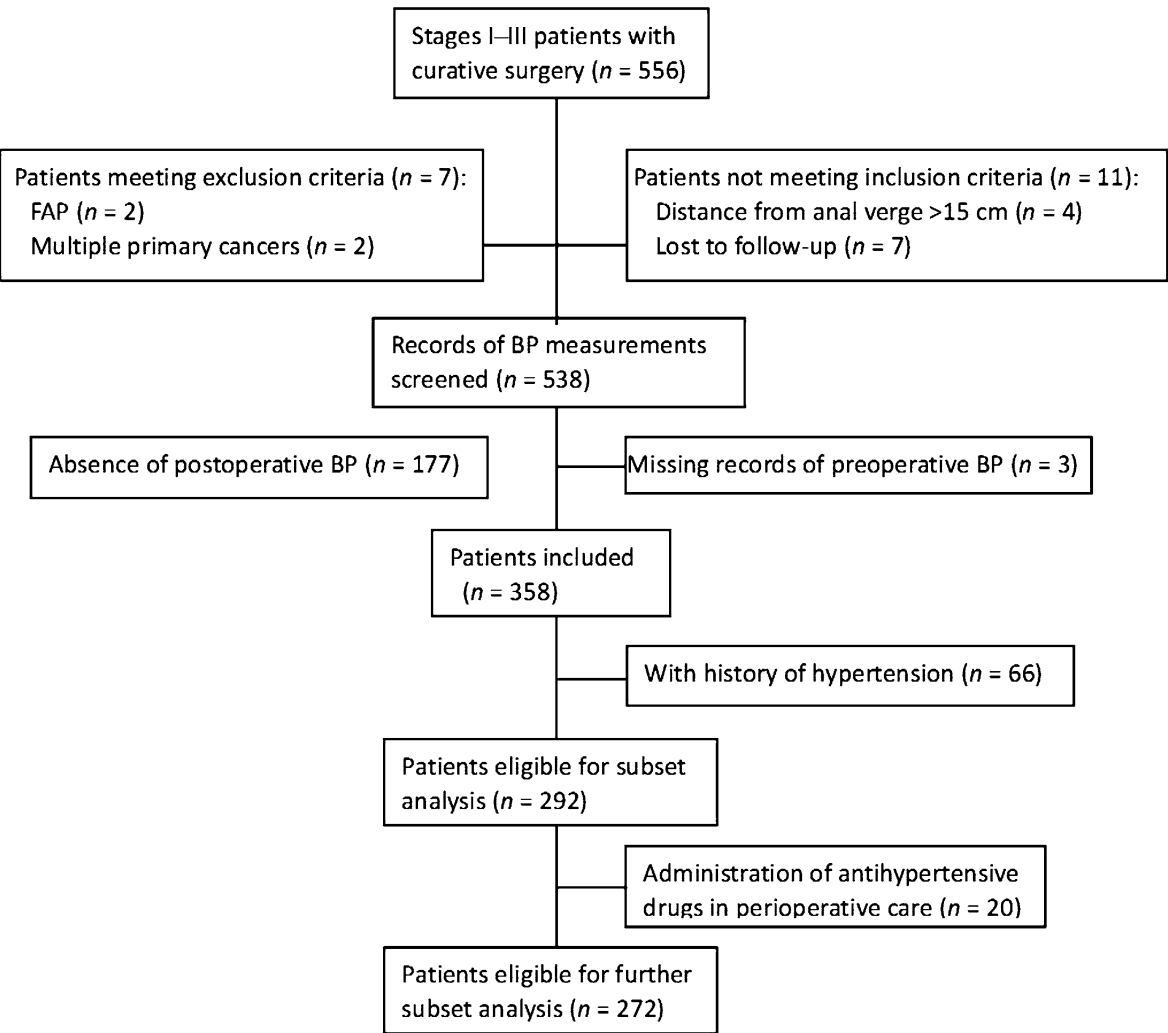
Flow diagram of patient selection. This diagram presents case disposition in the analysis of the association between perioperative BP and long-term survival in patients with rectal cancer. *BP* blood pressure, *FAP* familial adenomatous polyposis

We reviewed 556 patients with stages I–III rectal cancer who underwent radical surgery at our institution between June 2007 and June 2011. Rectal cancer was defined as histologically proven adenocarcinoma within 15 cm of the anal verge and was staged according to the 7th edition of the American Joint Committee on Cancer (AJCC) staging system [13], with staging procedures, including colonoscopy, contrast-enhanced computed tomography (CT) scans, and magnetic resonance imaging, performed at the initial diagnosis in all cases. The exclusion criteria were as follows: (1) absence of postoperative BP measured at 6:00 a.m. on two consecutive postoperative days; (2) familial adenomatous polyposis; and (3) multiple primary cancers. Of the 556 patients, 198 were excluded. Finally, this retrospective and longitudinal cohort study included 358 patients eligible for analysis (Fig. 1).

Demographic data (including age, height, and weight), tumor location, tumor staging, the tumor’s histologic features, blood transfusion, preoperative serum carcinoembryonic antigen (CEA) level, treatment regimen, time-to-recurrence, and cancer-specific survival (CSS) were collected from the Institutional Cancer Database and the Department of Inpatient Medical Records. The study was approved by the Medicine Ethics Committee of the Six Affiliated Hospital at Sun Yat-sen University.

### Measurement of body mass index and BP

For all patients, weight was measured in light indoor clothing, and height was measured without shoes. According to the World Health Organization recommendation for Asians based on obesity grade, body mass index (BMI) was categorized as underweight (BMI < 18.5 kg/m^2^), normal (BMI 18.5–23 kg/m^2^), and overweight-obese (BMI ≥ 23 kg/m^2^) [14]. Patients’ preoperative BP was measured when they were admitted to the hospital. Postoperative BP was defined as the mean value of two BPs measured at 6:00 a.m. on 2 consecutive postoperative days (days 1 and 2). Resting time before BP measurement ranged from 10 to 20 min, body position was sitting or supine, and the equipment used was a mercury sphygmomanometer or an automatic device. Hazard ratios (HRs) for long-term survival were calculated by levels of systolic blood pressure (SBP), diastolic blood pressure (DBP), and mid-BP [(SBP + DBP)/2]; SBP was also assessed in each subset by sex, BMI, and hypertension history. Mid-BP was primarily used because it is a strong predictor of cardiovascular mortality [15]. This measure, equally weighted for SBP and DBP, is considered an alternatively reasonable choice in the absence of knowledge about BP predictors in relation to rectal cancer. Pre- and postoperative BP results were classified using the Joint National Committee-7 definition of hypertension stage (BP: low <120/80 mmHg; high ≥120/80 mmHg) [16]. All patients were grouped according to these classifications.

### Treatment

Details of chemoradiotherapy were described in our previous publication [17]. Among the 358 patients included in this study, 43 (12%) received neoadjuvant chemoradiotherapy, in which a total irradiation dose of 46 Gy in 23 fractions of 2 Gy with concomitant application of 5-fluorouracil was given. At a median interval of 10.9 weeks (range, 6.9–17.3 weeks) after completion of chemoradiotherapy, total mesorectal excision (TME) for rectal cancer was implemented. Surgical techniques included low anterior resection, abdominoperineal resection, and the Parks procedure. Perioperative assessment of TME quality was performed on all surgical specimens. Based on physician suggestions and patient decisions, patients received 6–8 cycles of postoperative 5-fluorouracil-based chemotherapy, with a median interval of 4.4 weeks (range, 3.0–5.0 weeks).

### Follow-up

All patients were re-evaluated every 3 months for the first 3 years after surgery, every 6 months for the next 2 years, and yearly thereafter. Each evaluation included a pertinent medical history, a physical examination (including a rectal examination), and measurement of serum CEA concentration. Routine radiologic examinations consisting of chest radiography, abdominopelvic CT or ultrasonography, whole-body bone scintigraphy, and colonoscopy or double-contrast barium enema were performed 6 months after surgery and annually thereafter. Cancer recurrence was detected by CEA > 5 ng/mL and/or a sequential CT scan with evidence of disease followed by histopathologic confirmation. The primary endpoints of this study were disease-free survival (DFS) and CSS. DFS was defined as the time from surgery until recurrence; CSS was defined as the time from surgery to death from rectal cancer. The designated endpoint date was July 2014, with the interval of follow-up ranging from 3 to 7 years. Patients were censored at their last follow-up if the endpoint of interest had not been met.

### Statistical analyses

BPs were evaluated as both continuous and categorical variables using the median value. The intergroup comparisons of clinicopathologic variables were performed using the analysis of variance and Kruskal–Wallis tests for continuous variables (depending on the distribution of the continuous variables) and the Chi square and two-tailed Fisher’s exact tests for discrete variables. Postoperative survival was estimated using the Kaplan–Meier method. A univariate screening of potential risk factors of mortality using the Cox proportional hazards model for each variable extracted from medical records was performed. All significant risk factors in the univariate analysis were included in the multivariate analyses using the Cox proportional hazards model to identify independent risk factors. SBP, DBP, and mid-BP were never analyzed in the same model because of their multicollinearity. Data analysis was performed using SPSS version 22.0 software for Windows (SPSS, Inc., Chicago, IL, USA). All tests were two-sided, and *P* < 0.05 were considered statistically significant.

## Results

### Baseline characteristics

Baseline demographic and clinicopathologic data of the entire cohort and each group by postoperative SBP are summarized in Table 1. Of the 358 patients included in this study, 204 were men and 154 were women, with a median age of 60 years (range, 21–89 years). Of the 358 patients, 199 (55.6%) were categorized as underweight or normal weight, 135 (37.7%) were categorized as overweight or obese, and 24 (6.7%) had unknown BMI. In the entire cohort, 66 patients (18.4%) had a history of hypertension and long-term regular use of antihypertensive drugs; in the preoperative evaluation, 72 (20.1%) were categorized as having systolic hypertension (SBP ≥ 140 mmHg), 171 (47.8%) as having systolic prehypertension (SBP = 120–140 mmHg), 107 (29.9%) as having normal SBP (SBP < 120 mmHg), and 8 (2.2%) had unknown BP. The postoperative SBP, DBP, and mid-BP were statistically distributed with medians of 109.5 (80.0–171.0), 65.0 (42.5–102.0), and 87.5 (64.5–122.3) mmHg, respectively. The AJCC staging of rectal cancer in the entire cohort was distributed as 26.0%, 32.1%, and 41.9% for stage I, II, and III, respectively. No statistically significant differences were found between patients with postoperative SBP < 120 and ≥ 120 mmHg regarding demographic, morphometric, therapeutic regimen, and tumor characteristics; however, statistically significant differences were found in preoperative hypertension (42.0% vs. 11.6%, *P* < 0.001) and history of hypertension (41.0% vs. 9.7%, *P* < 0.001) between the high-postoperative SBP group and the low-postoperative SBP group.

**Table 1:** Baseline characteristics and distribution of clinicopathologic variables for the 358 patients with rectal cancer

Variable	Overall population	Postoperative SBP (mmHg)		*P* value
		<120	≥120	
	(*n* = 358)	(*n* = 258)	(*n* = 100)	
Age (years)				
Median (range)^a^	60 (21–89)	58 (21–89)	65 (25–89)	<0.001
<60	174 (48.6)	143 (55.4)	31 (31.0)	<0.001
≥60	184 (51.4)	115 (44.6)	69 (69.0)	
BMI (kg/m^2^)				
Median (range)^a^	22.1 (13.3–35.9)	22.0 (13.3–33.8)	22.7 (14.9–35.9)	0.080
<23	199 (55.6)	152 (58.9)	47 (47.0)	0.219
≥23	135 (37.7)	95 (36.8)	40 (40.0)	
Unknown	24 (6.7)	11 (4.3)	13 (13.0)	
Sex				0.631
Men	204 (57.0)	145 (56.2)	59 (59.0)	
Women	154 (43.0)	113 (43.8)	41 (41.0)	
History of hypertension				<0.001
No	292 (81.6)	233 (90.3)	59 (59.0)	
Yes	66 (18.4)	25 (9.7)	41 (41.0)	
History of smoking				0.001
No	263 (73.5)	202 (78.3)	61 (61.0)	
Yes	95 (26.5)	56 (21.7)	39 (39.0)	
Preoperative BP (mmHg)				
Median (range)^a^	123 (73–201)	120 (73–184)	135 (97–201)	<0.001
<120	107 (29.9)	94 (36.4)	13 (13.0)	<0.001
120–140	171 (47.8)	130 (50.4)	41 (41.0)	
≥140	72 (20.1)	30 (11.6)	42 (42.0)	
Unknown	8 (2.2)	4 (1.6)	4 (4.0)	
TNM stage, AJCC				0.537
I	93 (26.0)	71 (27.5)	22 (22.0)	
II	115 (32.1)	80 (31.0)	35 (35.0)	
III	150 (41.9)	107 (41.5)	43 (43.0)	
Differentiation degree				0.353
Low	68 (19.0)	47 (18.2)	21 (21.0)	
Moderate	188 (52.5)	132 (51.2)	56 (56.0)	
High	102 (28.5)	79 (30.6)	23 (23.0)	
Vascular invasion				0.782
Negative	327 (91.3)	235 (91.1)	92 (92.0)	
Positive	31 (8.7)	23 (8.9)	8 (8.0)	
Perineural invasion				0.698
Negative	326 (91.1)	234 (90.7)	92 (92.0)	
Positive	32 (8.9)	24 (9.3)	8 (8.0)	
Preoperative CEA				0.382
Negative	252 (70.4)	185 (71.7)	67 (67.0)	
Positive	106 (29.6)	73 (28.3)	33 (33.0)	
Distance from anal verge (cm)				0.831
<5	132 (36.9)	96 (37.2)	36 (36.0)	
5–12	226 (63.1)	162 (62.8)	64 (64.0)	
Intraoperative blood transfusion				0.514
No	311 (86.9)	226 (87.6)	85 (85.0)	
Yes	47 (13.1)	32 (12.4)	15 (15.0)	
Estimation of blood loss in operation [median (range); mL]^a^	100 (15–3000)	100 (20–3000)	100 (15–1000)	0.899
Adjuvant treatment				0.122
No	177 (49.4)	121 (46.9)	56 (56.0)	
Yes	181 (50.6)	137 (53.1)	44 (44.0)	
Neoadjuvant treatment				0.146
No	315 (88.0)	223 (86.4)	92 (92.0)	
Yes	43 (12.0)	35 (13.6)	8 (8.0)	

*SBP* systolic blood pressure, *BMI* body mass index, *BP* blood pressure, *AJCC* American Joint Committee on Cancer, *CEA* carcinoembryonic antigen

^a^These values are presented as median followed by a range in the parentheses; other values are presented as the number of cases with a percentage in the following parentheses

### Univariate and multivariate analyses of survival

**Fig. 2:**
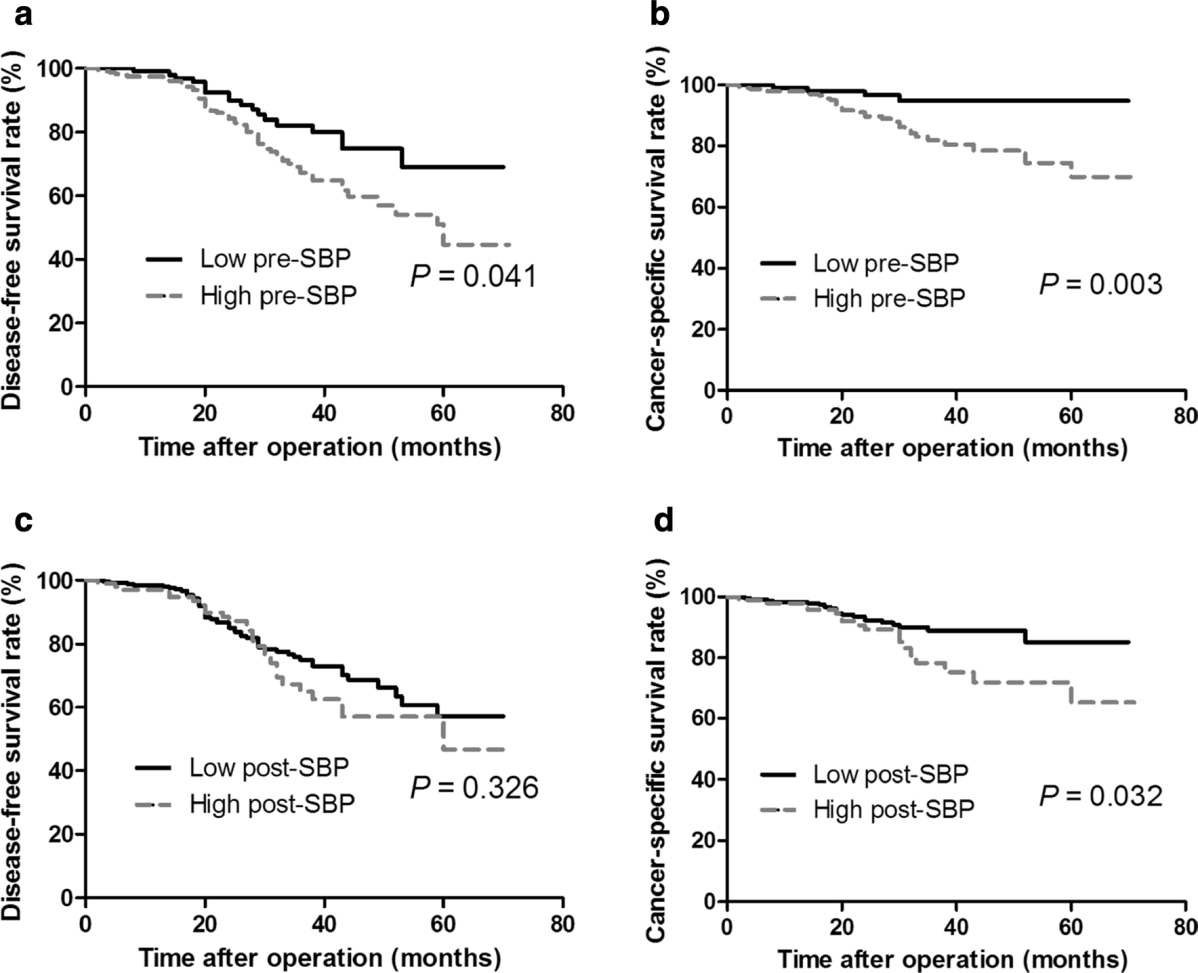
Kaplan–Meier survival curves of rectal cancer patients with different preoperative and postoperative BPs. The *curves* showed significantly lower DFS (**a**) and CSS (**b**) rates in the high-preoperative SBP group than in the low-preoperative SBP group. Patients in the high-postoperative SBP group had significantly lower CSS (**d**) rates, whereas the differences in the DFS curves (**c**) were not significant. *SBP* systolic blood pressure, *DFS* disease-free survival, *CSS* cancer-specific survival

Median follow-up time was 42 months (range, 37–85 months). Kaplan–Meier curves showed significantly lower 3-year DFS rate (67.2% vs. 82.1%, *P* = 0.041) and CSS rate (81.9% vs. 94.8%, *P* = 0.003) in the high-preoperative SBP group than in the low-preoperative SBP group. Similarly, patients in the high-postoperative SBP group had a significantly lower 3-year CSS rate (78.3% vs. 88.9%, *P* = 0.032) than patients in the low-postoperative SBP group, whereas the difference in the DFS rate (65.1% vs. 75.0%, *P* = 0.326) was not significant (Fig. 2).

Univariate analysis showed that old age, advanced AJCC stage, vascular invasion, perineural invasion, elevated CEA level, low rectal cancer, and high pre- and postoperative BP were all significantly associated with increased mortality (Tables 2, 3). Specifically, HRs for BP and survival for total cases and for patients in each subset are shown in Table 3. In preoperative evaluation, patients with high SBP had lower 3-year DFS and CSS rates compared with patients with low SBP, the HRs of which were 1.87 (95% CI, 1.11–3.17, *P* = 0.019) and 4.18 (95% CI, 1.48–11.83, *P* = 0.007), respectively; in postoperative evaluation, these HRs for SBP were 1.26 (95% CI, 0.79–1.98, *P* = 0.330) and 1.99 (95% CI, 1.05–3.78, *P* = 0.035), respectively. The HR of the CSS rate for postoperative mid-BP was 2.34 (95% CI 1.16–4.72, *P* = 0.018).

**Table 2:** Univariate analysis of prognostic factors for DFS and CSS

Factor	DFS			CSS		
	3-year rate (%)	HR (95% CI)	*P*	3-year rate (%)	HR (95% CI)	*P*
Age	–	1.00 (0.99–1.02)	0.644	–	1.03 (1.01–1.06)	0.016
TNM stage, AJCC						
III	60.3	3.94 (1.95–7.99)	<0.001	79.7	5.24 (1.58–17.42)	0.007
II	79.7	1.81 (0.82–4.01)	0.142	86.6	3.14 (0.88–11.24)	0.079
I	84.4	1.00		94.7	1.00	
Vascular invasion						
Positive	59.3	1.93 (1.10–3.39)	0.021	77.6	1.70 (0.71–4.07)	0.234
Negative	73.7	1.00		86.7	1.00	
Perineural invasion						
Positive	38.0	3.44 (1.98–5.98)	<0.001	73.8	2.45 (1.02–5.90)	0.046
Negative	75.4	1.00		87.0	1.00	
Preoperative CEA (ng/mL)						
>5	63.9	1.59 (1.00–2.52)	0.050	75.2	2.91 (1.48–5.71)	0.002
0–5	77.2	1.00		92.2	1.00	
Distance from anal verge (cm)						
5–12	75.5	0.59 (0.37–0.92)	0.021	89.3	0.47 (0.24–0.92)	0.028
<5	69.3	1.00		82.0	1.00	
Adjuvant treatment						
Yes	77.6	0.76 (0.49–1.18)	0.226	90.0	0.49 (0.25–0.94)	0.031
No	67.2	1.00		80.7	1.00	

*DFS* disease-free survival, *CSS* cancer-specific survival, *HR* hazard ratio, *CI* confidence interval, *SBP* systolic blood pressure, *BMI* body mass index, *BP* blood pressure, *AJCC* American Joint Committee on Cancer, *CEA* carcinoembryonic antigen

**Table 3:** Univariate analysis of the association between BPs and survival outcome

Variable	Blood pressure	No. of patients in low BP group/high BP group	DFS					CSS				
			Categorized, HR (95% CI)			Continuous HR (95% CI)	*P*	Categorized, HR (95% CI)			Continuous HR (95% CI)	*P*
			<Cut-off (ref)	≥Cut-off	*P*			<Cut-off (ref)	≥Cut-off	*P*		
Total	Preoperative BP	358										
	SBP	111/247	1.00	1.87 (1.11–3.17)	0.019	1.01 (0.99–1.01)	0.229	1.00	4.18 (1.48–11.83)	0.007	1.00 (0.99–1.01)	0.726
	DBP	207/151	1.00	1.44 (0.93–2.23)	0.107	1.01 (0.99–1.03)	0.221	1.00	1.72 (0.89–3.31)	0.106	1.00 (0.98–1.02)	0.971
	Mid-BP	161/197	1.00	1.54 (0.98–2.42)	0.060	1.01 (0.99–1.03)	0.121	1.00	1.73 (0.87–3.42)	0.117	1.02 (0.99–1.04)	0.145
	Postoperative BP	358										
	SBP	258/100	1.00	1.26 (0.79–1.98)	0.330	1.00 (0.99–1.02)	0.807	1.00	1.99 (1.05–3.78)	0.035	1.01 (0.99–1.03)	0.207
	DBP	337/21	1.00	1.33 (0.61–2.88)	0.477	1.01 (0.98–1.03)	0.625	1.00	2.15 (0.84–5.53)	0.111	1.03 (0.99–1.06)	0.106
	Mid-BP	306/52	1.00	1.63 (0.96–2.74)	0.069	1.00 (0.99–1.02)	0.716	1.00	2.34 (1.16–4.72)	0.018	1.02 (0.99–1.05)	0.131
Sex												
Men	Preoperative SBP	62/135	1.00	1.89 (0.90–3.97)	0.091	1.00 (0.99–1.01)	0.701	1.00	3.37 (1.00–11.39)	0.050	1.00 (0.99–1.01)	0.777
	Postoperative SBP	145/59	1.00	1.35 (0.73–2.50)	0.334	1.00 (0.98–1.02)	0.957	1.00	1.76 (0.79–3.93)	0.170	1.01 (0.98–1.03)	0.450
Women	Preoperative SBP	45/108	1.00	1.79 (0.85–3.78)	0.128	1.01 (0.99–1.02)	0.230	1.00	6.16 (0.81–47.19)	0.080	1.02 (0.99–1.04)	0.191
	Postoperative SBP	113/41	1.00	1.25 (0.62–2.52)	0.531	1.01 (0.99–1.03)	0.509	1.00	2.32 (0.80–6.69)	0.120	1.02 (0.98–1.05)	0.380
History of hypertension												
Yes	Preoperative SBP	5/61	1.00	1.13 (0.37–3.49)	0.350	1.00 (0.99–1.02)	0.665	1.00	0.85 (0.17–4.41)	0.848	1.00 (0.98–1.02)	0.959
	Postoperative SBP	25/41	1.00	0.56 (0.20–1.56)	0.269	0.99 (0.96–1.03)	0.746	1.00	0.40 (0.09–1.79)	0.229	0.97 (0.92–1.02)	0.180
No	Preoperative SBP	102/183	1.00	1.66 (0.96–2.85)	0.070	1.00 (0.99–1.01)	0.412	1.00	3.84 (1.34–11.05)	0.012	1.00 (0.99–1.02)	0.804
	Postoperative SBP	233/59	1.00	1.36 (0.80–2.31)	0.255	0.99 (0.98–1.02)	0.945	1.00	2.77 (1.36–5.66)	0.005	1.02 (1.00–1.05)	0.053
BMI												
BMI <23	Preoperative SBP	70/125	1.00	1.42 (0.75–2.69)	0.283	1.00 (0.99–1.02)	0.820	1.00	2.40 (0.79–7.24)	0.120	0.99 (0.98–1.01)	0.352
	Postoperative SBP	152/47	1.00	1.79 (0.96–3.33)	0.066	1.01 (0.99–1.03)	0.246	1.00	2.69 (1.13–6.38)	0.025	1.02 (0.99–1.04)	0.129
BMI ≥23	Preoperative SBP	29/104	1.00	1.99 (0.69–5.70)	0.201	1.00 (0.99–1.01)	0.709	1.00	1.08 (0.23–5.00)	0.247	1.01 (0.99–1.02)	0.636
	Postoperative SBP	95/40	1.00	0.70 (0.31–1.58)	0.394	0.98 (0.95–1.01)	0.112	1.00	1.01 (0.30–3.36)	0.987	0.99 (0.95–1.03)	0.561

*DBP* diastolic blood pressure, *DFS* disease-free survival, *CSS* cancer-specific survival, *HR* hazard ratio, *CI* confidence interval, *SBP* systolic blood pressure, *BMI* body mass index, *BP* blood pressure, *AJCC* American Joint Committee on Cancer, *CEA* carcinoembryonic antigen

In multivariate analysis, old age, advanced AJCC stage, perineural invasion, low rectal cancer, adjuvant treatment regimen, and high preoperative SBP (adjusted HR = 1.97, 95% CI = 1.08–3.60, *P* = 0.028) were still significantly associated with 3-year DFS rate. Advanced AJCC stage, elevated preoperative CEA level, low rectal cancer, adjuvant treatment regimen, and high preoperative SBP (adjusted HR = 2.85, 95% CI = 1.00–8.25, *P* = 0.050) had independently significant prediction value on 3-year CSS. Nevertheless, postoperative SBP did not remain significant in the model for either DFS or CSS (Table 4).

**Table 4:** Multivariate analysis of prognostic factors for DFS and CSS

Variable	HR (95% CI)	*P*
DFS		
Age	0.98 (0.96–0.99)	0.039
AJCC stage III	5.71 (2.44–13.37)	<0.001
Perineural invasion	2.17 (1.09–4.32)	0.028
Distance from anal verge ≥5 cm	0.53 (0.32–0.85)	0.009
Adjuvant treatment	0.41 (0.23–0.71)	0.002
Preoperative SBP ≥120 mmHg	1.97 (1.08–3.60)	0.028
CSS		
AJCC stage III	7.45 (1.65–33.64)	0.009
Preoperative CEA >5 ng/mL	2.18 (1.03–4.61)	0.042
Distance from anal verge ≥5 cm	0.36 (0.17–0.77)	0.009
Adjuvant treatment	0.36 (0.17–0.78)	0.010
Preoperative SBP ≥120 mmHg	2.85 (1.00–8.25)	0.050

*DFS* disease-free survival, *CSS* cancer-specific survival, *HR* hazard ratio, *CI* confidence interval, *SBP* systolic blood pressure, *BMI* body mass index, *BP* blood pressure, *AJCC* American Joint Committee on Cancer, *CEA* carcinoembryonic antigen

### Analyses on subsets of patients without history of hypertension

Further subset analyses were conducted to determine if the above association between perioperative BP and survival depends on the long-term negative effect of patients’ history of hypertension on tumorigenesis and cancer mortality. For 292 patients without a history of hypertension, the HRs of CSS for pre- and postoperative SBP were 3.84 (95% CI, 1.34–11.05, *P* = 0.012) and 2.77 (95% CI, 1.36–5.66, *P* = 0.005), respectively, whereas these associations were not significant in patients with a history of hypertension (Table 3). To further validate if the verified association with cancer risk depends on the negative effect of antihypertensive drugs, we excluded 86 patients with a history of hypertension and/or perioperative administration of antihypertensive drugs (Fig. 1); the survival outcomes were compared between the low-SBP group and the high-SBP group. In the analyses of the remaining 272 patients, the HRs were 3.36 (95% CI, 1.15–9.84, *P* = 0.027) and 2.15 (95% CI, 0.71–3.39, *P* = 0.076), respectively.

Other associations of SBPs were analyzed for DFS and/or CSS among the subsets by sex and BMI (Table 3). In men, the HR of CSS for postoperative SBP was 3.37 (95% CI, 1.00–11.39, *P* = 0.050), whereas in women, the association was not significant. Interestingly, neither pre- nor postoperative SBP was significantly associated with survival in 135 patients with a BMI ≥ 23 kg/m^2^.

## Discussion

Our study showed that perioperative BP is negatively associated with survival outcome in patients with rectal cancer. In agreement with our hypothesis, 3-year CSS was associated with high pre- and postoperative BP in patients with rectal cancer treated with radical surgery, and high preoperative SBP was an independent risk factor. These findings were confirmed after we stratified patients and only looked at the tumors of patients without a history of hypertension.

The role of high BP as a possible risk factor for cancer mortality was first proposed in 1975 by Dyer et al. [10] and has since been examined in various cohort studies. In most studies, BP was measured at the time of the baseline health examination when patients were enrolled, and to date only Park’s study—on renal carcinoma—addressed the association between perioperative BP and mortality [18]. However, the investigators examined the association between perioperative BP and renal carcinoma mortality without stratification by history of hypertension and adjustment for some potential confounding factors. Since it is well established that hypertension has an unfavorable effect on tumorigenesis and cancer mortality over a long period of time and that a history of hypertension is positively linked with perioperative BP, Park’s study was not able to determine the association between perioperative BP and cancer risk. In our analyses on subsets of patients without a history of hypertension, the remained association between high perioperative SBP and CSS risk indicated that the efforts to control BP in perioperative care may extend survival for this population. However, we did not exclude the possibility that undiagnosed hypertension and hypertension developed after the surgery affected our results. Moreover, the targets for BP control should be determined in a larger cohort.

Many large trials have provided unassailable evidence that antihypertensive therapy lowers cardiovascular morbidity and mortality [19–21]. However, not all studies have demonstrated a significant association between antihypertensive treatment and overall mortality [22]; indeed, others have reported that the association is diminished over time [20]. Since then, many studies continued to report a putative link between antihypertensive drugs and cancer, with diuretics being the most frequently cited suspect. These findings thus prompt the question: is high BP associated with increased cancer mortality, or is the use of antihypertensive therapy implicated? After excluding the patients with a history of hypertension and perioperative administration of antihypertensive drugs, the analyses were repeated in our study. The results remained virtually unchanged. Since our results from a series of subset analyses suggested cancer mortality is associated with perioperative high BP, diuretics could be substituted for other classes of antihypertensive drugs, although diuretics are currently recommended as the first-line treatment for patients with hypertension [23].

The biological mechanisms that could account for the association between hypertension and elevated cancer mortality remain unclear. Studies have suggested that hypertension and malignancy might share several common biochemical pathways leading to proliferative abnormalities in vascular smooth muscle cells [24], increased level of inositol triphosphate and cytosolic calcium [25], and abnormalities in carcinogen binding to DNA in lymphocytes [26], which are associated with both hypertension and tumorigenesis. Furthermore, factors related to hypertension, such as age, smoking, and alcohol consumption, might contribute to increased cancer mortality. However, these hypotheses and findings could not account for the involvement of high perioperative SBP that is independent of a history of hypertension in cancer mortality. Therefore, we hypothesized that surgical procedures may augment the release of cancer cells into circulation [27], and then high BP in the perioperative period might enable the circulating cancer cells to penetrate blood vessels, which partially contributes to local recurrence, distant metastasis, and cancer-specific death.

Our results indicate that the cancer-specific death risk of high SBP is higher and statistically significant in men and in patients with a low BMI, which is consistent with the findings from previous study of all-site cancer and colorectal cancer [6]. Male sex, BMI, elevated perioperative BP, and hypertension can be related, with a possibility of a more complex pattern among these factors in the risk of cancer-specific death.

Nevertheless, our study does have limitations. First, regarding the subgroup analyses, we were not able to determine the patients’ precise overall use of antihypertensive drugs; therefore, the quantification of risks as presented may not be valid. Second, this was a retrospective study and thus was limited by unknown confounders owing to its observational nature, although some variables were controlled. Although BP was predictive in our study, future studies in larger or prospective cohorts are necessary to validate the association.

## Conclusions

This study showed that high preoperative SBP was an independent risk factor for both CSS and DFS and that high postoperative SBP was significantly associated with poor CSS. Furthermore, our subset analyses suggested that the unfavorable effect of high BP is independent of a patient’s history of hypertension and use of antihypertensive drugs. Additionally, our results suggest that patients with rectal cancer may get survival benefit from BP control in perioperative care. However, future studies should determine this association and identify the targets of BP control.
